# Development of a transmission model for dengue virus

**DOI:** 10.1186/1743-422X-10-127

**Published:** 2013-04-23

**Authors:** Rebecca C Christofferson, Michael K McCracken, Ann-Marie Johnson, Daniel M Chisenhall, Christopher N Mores

**Affiliations:** 1 Department of Pathobiological Sciences, School of Veterinary Medicine, Louisiana State University, Skip Bertman Drive, Baton Rouge, LA 70803, USA

**Keywords:** Dengue, Transmission, Arbovirus, Mouse

## Abstract

**Background:**

Dengue virus (DENV) research has historically been hampered by the lack of a susceptible vertebrate transmission model. Recently, there has been progress towards such models using several varieties of knockout mice, particularly those deficient in type I and II interferon receptors. Based on the critical nature of the type I interferon response in limiting DENV infection establishment, we assessed the permissiveness of a mouse strain with a blunted type I interferon response via gene deficiencies in interferon regulatory factors 3 and 7 (IRF3/7 ^−/− −/−^) with regards to DENV transmission success. We investigated the possibility of transmission to the mouse by needle and infectious mosquito, and subsequent transmission back to mosquito from an infected animal during its viremic period.

**Methods:**

Mice were inoculated subcutaneously with non-mouse adapted DENV-2 strain 1232 and serum was tested for viral load and cytokine production each day. Additionally, mosquitoes were orally challenged with the same DENV-2 strain via artificial membrane feeder, and then allowed to forage or naïve mice. Subsequently, we determined acquisition potential by allowing naïve mosquitoes on forage on exposed mice during their viremic period.

**Results:**

Both needle inoculation and infectious mosquito bite(s) resulted in 100% infection. Significant differences between these groups in viremia on the two days leading to peak viremia were observed, though no significant difference in cytokine production was seen. Through our determination of transmission and acquisition potentials, the transmission cycle (mouse-to mosquito-to mouse) was completed. We confirmed that the IRF3/7 ^−/− −/−^ mouse supports DENV replication and is competent for transmission experiments, with the ability to use a non-mouse adapted DENV-2 strain. A significant finding of this study was that this IRF3/7 ^−/− −/−^ mouse strain was able to be infected by and transmit virus to mosquitoes, thus providing means to replicate the natural transmission cycle of DENV.

**Conclusion:**

As there is currently no approved vaccine for DENV, public health monitoring and a greater understanding of transmission dynamics leading to outbreak events are critical. The further characterization of DENV using this model will expand knowledge of key entomological, virological and immunological components of infection establishment and transmission events.

## Background

Dengue virus (DENV) is the most common arboviral infection of humans, infecting tens of millions of people each year in tropical areas [1]. There is presently no vaccine available for DENV and reducing transmission via mosquito control efforts has not yet been sufficient to alter the trajectory of the pandemic [2]. DENV is efficiently spread from mosquito to human and back through a domestic cycle that is easily described on a superficial level; however, numerous studies have suggested that our accounting of the factors involved in hyperendemic, community, and inter-annual transmission, among others, remains poorly characterized. It is widely agreed that enhancing our understanding of these variable transmission determinants is vital to controlling outbreaks [3-6]. However, characterization of these factors and their relative importance on transmission has been complicated by the lack of a suitable laboratory model for DENV transmission.

**Figure 1:**
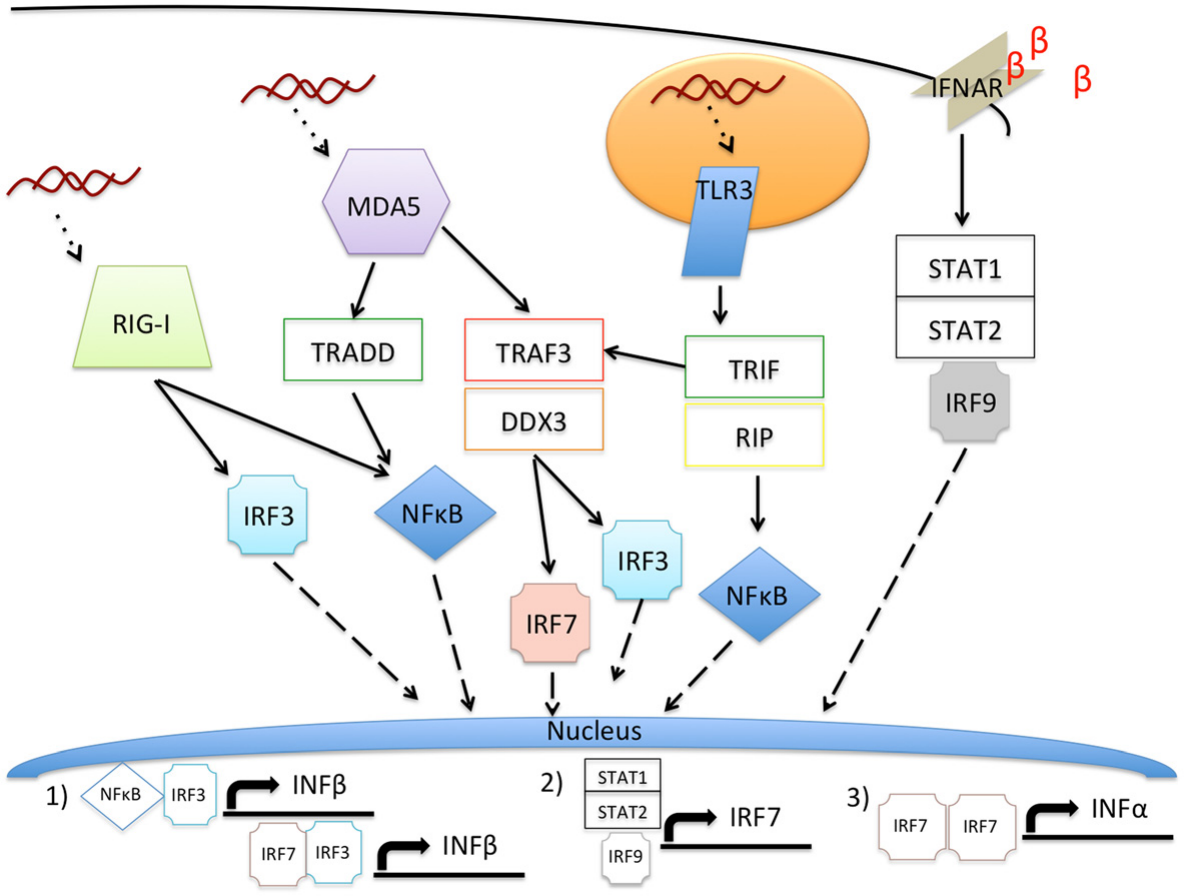
**Type I interferon production signals through IRF3 and IRF7.** Double stranded RNA is recognized by RIG-I and MDA5 in the cytoplasm and double stranded RNA is recognized in the endosome by TLR3. The resulting signal cascades all utilize IRF 3 and 7 to first promote transcription of IFN-β, which positively feeds back via STAT1/STAT2/IRF9 to promote transcription of IRF7, which promotes transcription of IFN-α.

Immunologically intact mouse models have been shown to be resistant to DENV infections, due to the ability of their innate immune responses to quickly and efficiently clear the virus, though success has been seen with mouse-adapted viruses and/or artificial infection routes such as intracranial and intraperitoneal injection [7-13]. The innate immune response certainly engages DENV extracellularly, but once DENV infiltrates a cell via receptor-mediated endocytosis, (retinol inducible gene-1) RIG-I is stimulated by the presence of double stranded viral RNA (vRNA). Additionally, melanoma differentiation-associated protein (MDA5) and endosomal Toll-like receptor (TLR) -3 recognize double stranded vRNA intermediates generated during viral replication. These pathways signal through the interferon regulatory factors (IRF) 3 and 7, which initiate a cascade of signals resulting in the transcription of type I interferon (IFN-α and -β) [10]. Figure 1 demonstrates the importance of IRF3 and IRF7 in the type I IFN pathway. Briefly, through the activation of these pathways, (nuclear factor kappa-light-chain-enhancer of activated B cells) NFκB and IRF3 localize to the nucleus to promote transcription of IFN-β. IFN-β binds the type I IFN receptor (IFNAR), which leads to the localization of the (signal transducers and activators of transcription) STAT1/STAT2/IRF9 complex to the nucleus where IRF7 transcription is promoted. Up-regulated production of IRF7 leads to increased localization of IRF7 dimers to the nucleus where the production of IFN-α is promoted. IFN-α and -β are responsible for the establishment and maintenance of the anti-viral state in the immune response [8-10,14,15].

*In vitro* studies have shown either that DENV inhibits type I IFN production or that lack of type I IFN response renders mice susceptible, indicating that this mechanism of immune response subversion is critical for DENV fitness and thus affects transmission [16-18]. Further, others have shown that downstream protein expression induced by type I IFN and the (Janus kinase) JAK/STAT pathway play important roles in DENV inhibition [17-21]. Much attention has been given to this JAK/STAT pathway and it has been suggested that DENV viral mediated subversion of the type I IFN response occurs only after this pathway has been initiated [20,22].

Recently, there has been progress towards DENV vertebrate infection models based on these findings with successful studies involving several knockout mice, particularly the AG129 model deficient in type I and II interferon receptors and a more recent C57BL/6 mouse deficient in type I IFN receptors [18,23-29]. These animals have most often been used to model DENV disease and pathogenesis, especially the severe forms of disease [29-34]. Humanized mice have also shown great promise for investigating DENV pathogenesis as well as transmission [34,35]. This is the exception, however, as the use of many of these animal models for DENV transmission experiments has yet to be explored.

Accordingly, we assessed the permissiveness of a strain of C57BL/6 mouse lacking IRF3 and IRF7 (IRF3/7 ^−/− −/−^) with respect to the transmission of non-mouse adapted DENV [18,36-38]. Specifically, we investigated the possibility of non-mouse adapted DENV transmission to this mouse strain by needle inoculation or via infectious mosquito bite. Subsequently, we determined the potential for transmission back to mosquitoes from a viremic animal. Others have established the crucial role of type I IFN in early clearance of DENV, though none have sought to explore infection establishment [39]. In addition, some studies have indicated both types I and II IFN are necessary for disease progression [17,39-41]. Herein, we also investigated critical innate immune markers of progression, comparing infection establishment and kinetic differences from needle inoculation to that of *Aedes aegypti* mosquito transmission, as mosquito saliva and salivary gland proteins are known to affect pathogen transmission and/or disease progression [35,42-45].

## Results

### Permissive transmission model

**Figure 2:**
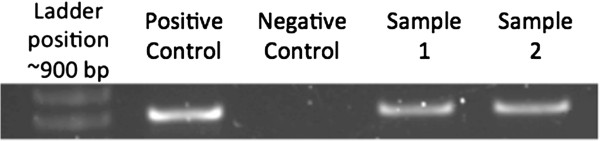
**Gel showing replicative strand amplification at 48 hours post inoculation with DENV-2 1232.** Left to Right: Ladder position (~900 bp), Positive control- Freeze thawed DENV-2 1232 infected Vero cell culture; Negative control- Uninfected, age matched Vero cell culture; Samples 1 and 2: Samples from inoculation site and draining lymph node of mice inoculated with DENV-2 1232, respectively.

**Figure 3:**
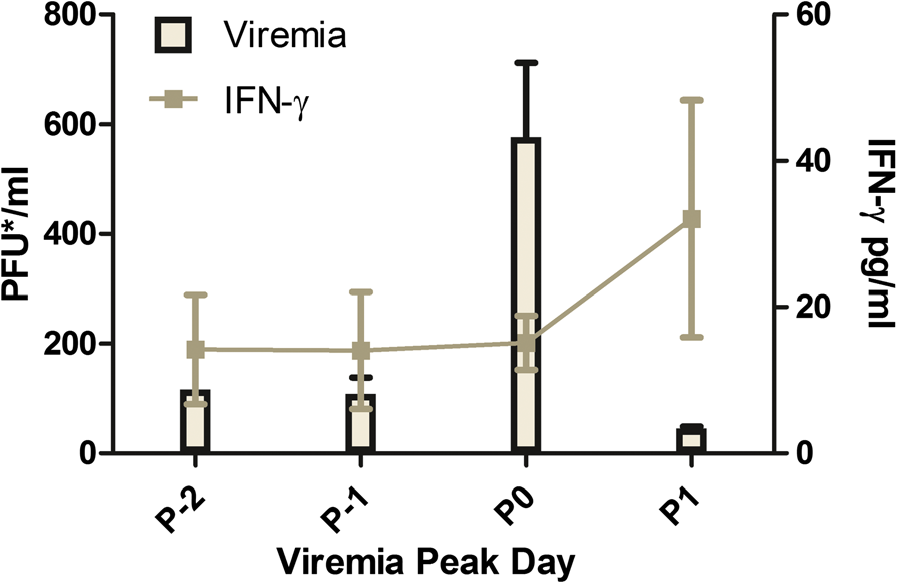
**Mean viremia and IFN-gamma levels of DENV-2 1232 needle inoculated mice.** Mean viremia and IFN-γ levels (+/− SEM) are centered around viremia peak day (peak = P0). IFN-γ levels peaked the day following peak viremia.

Mice (n = 2) used to detect the replicative strand of DENV vRNA had equivalent levels of viremia to the mice used in the analysis, but because they were sacrificed prior to their probable peak viremia day (48 hours post-infection), they were not included in the analyses here (Figure 2). Subsequent transmission of virus via subcutaneous needle inoculation to IRF3/7 ^−/− −/−^ mice was 100% successful (5/5 positive for DENV). Viremia levels were centered on the day of peak viremia (P0, Figure 3). In contrast, none of the DENV-2 inoculated wild type C57BL/6 mice (n = 5) developed detectable viremia at any time point (data not shown).

### Mosquito versus needle transmission

**Figure 4:**
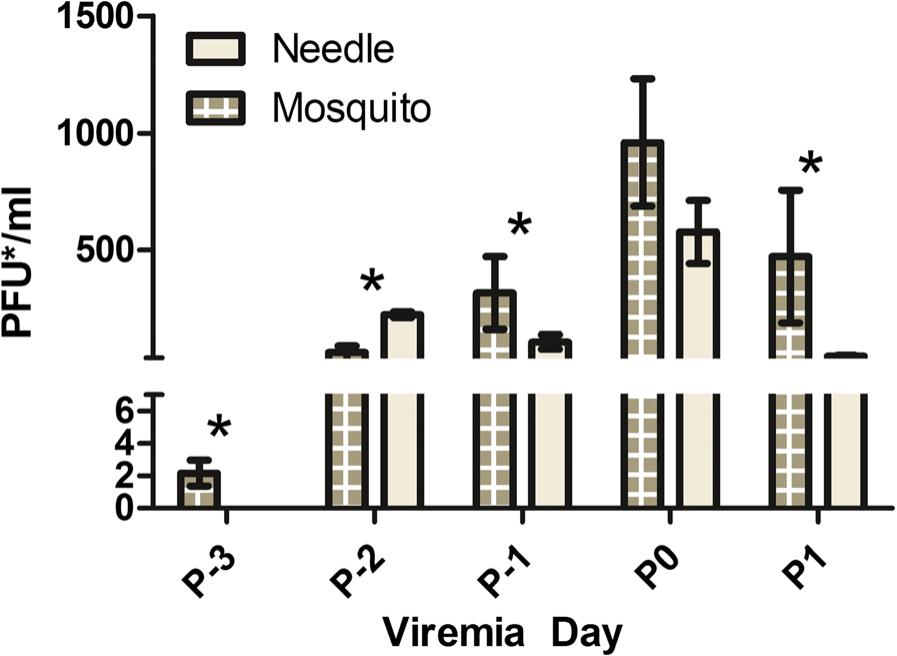
**DENV-2 1232 viremia levels of mosquito vs. needle inoculated mice.** Mean viremia (+/− SEM) in mosquito-inoculated mice was detectable for one day more than needle inoculated mice (5 days vs. 4 days, respectively). Viremia levels between the two treatment groups were statistically different (as indicated by *) at all times points except peak viremia day, P0.

To detect whether the responses to DENV transmission are different when IRF3/7 ^−/− −/−^ mice were exposed via infected mosquitoes, we exposed mice to mosquitoes that were previously orally challenged and later confirmed to have a disseminated DENV infection over two experiments (n = 7, n = 3). Of the ten mice exposed to infectious mosquitoes, all (10/10) developed viremia. Viremia levels in mosquito-exposed mice were statistically higher than those of the needle inoculated mice at all time points except peak viremia day, P0, and lasted one day longer (Figure 4). Viremia peaked one day later post-exposure in mosquito-exposed mice.

**Figure 5:**
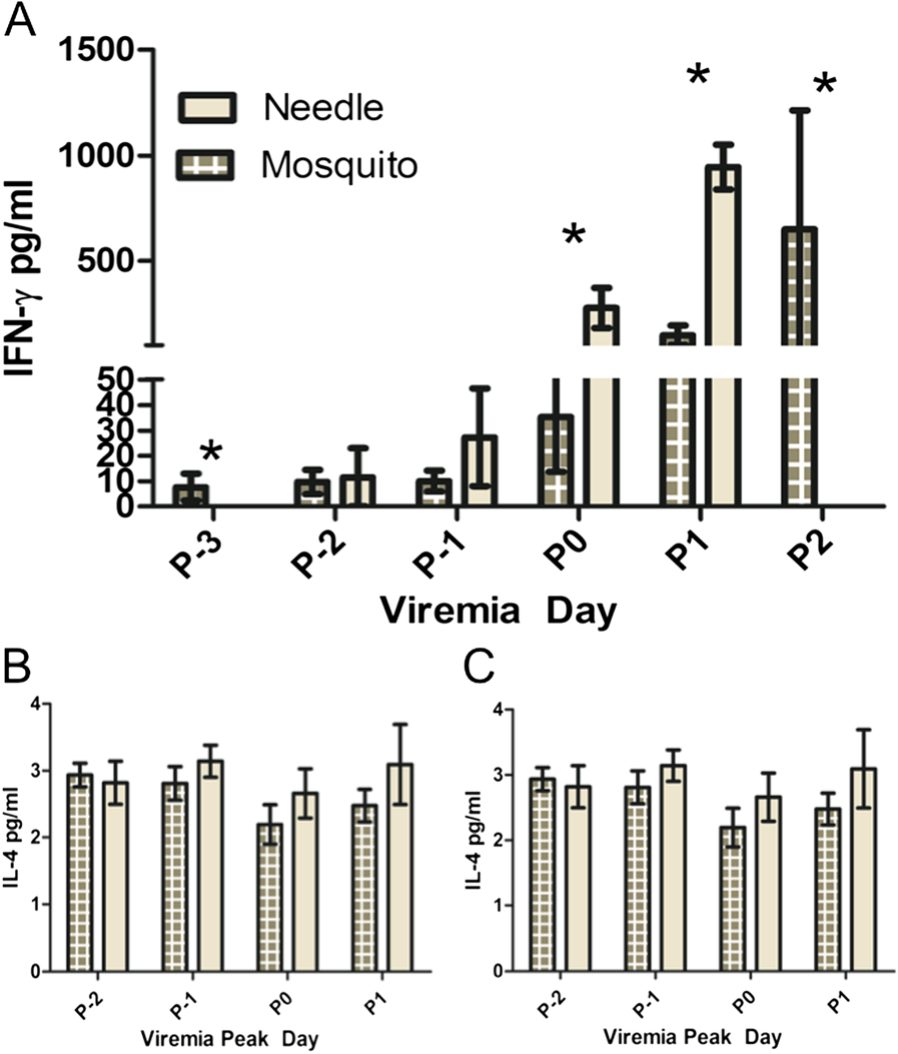
**Cytokine responses in needle-inoculated vs. mosquito-inoculated infections. A**) IFN-γ. The IFN-γ response (+/− SEM) was significantly higher in the needle inoculated group on days P0 and P1, as indicated by *. **B**) TNF-α. Means of serum TNF-α (+/− SEM) were not significantly different between mosquito and needle inoculated mice. **C**) IL-4. Means of serum IL-4 (+/−SEM) were not significantly different between mosquito and needle inoculated mice.

Because IFN-γ is a major difference between this and other currently used immune-deficient mouse models, we looked at the IFN-γ response to determine if it would be important in needle versus mosquito exposures. IFN-γ production rose noticeably in both the mosquito-exposed and needle-inoculated groups on P1 (Figure 5A). While the IFN-γ was measurable in the mosquito exposed mice one day earlier relative to peak day, the needle inoculated mice had significantly higher IFN-γ levels on P0 and P1. Additionally, the peak in IFN-γ in mosquito-exposed mice appears to be delayed to two days post peak viremia. In both groups, viremia was not detected following the peak of IFN-γ. Between the needle inoculated and mosquito-exposed mice, there was no significant difference in either the IL-4 or the TNF-α response (Figure 5B-C). In addition, in all groups, IL-1β was below the limit of detection.

### Kinetics of transmission and number of inoculating mosquitoes

We tested the hypothesis that the number of mosquitoes may affect transmission kinetics of mosquito-inoculated infections. The day of peak viremia and the viremia titer on P0 were not dependent on the number of infectious mosquitoes to which the mice were exposed (p > 0.05). The onset of viremia, however, was negatively associated with the number of infectious mosquitoes transmitting virus (regression slope = −.012, p < 0.05, Table 1), meaning that more mosquitoes translated into earlier onset of viremia. The average peak viremia was 7.14 × 10^2^ PFU/mL (min. 3.0 × 10^1^ PFU/mL, max. 2.15 × 10^3^ PFU/mL). These results are summarized in Table 1.

**Table 1:** **Summary of transmission from mosquito to IRF3/7**^**−/− −/− **^**mice**

**Mouse number**	**No. of infectious mosquitoes**	**Day of viremia onset (dpi)**	**Viremia**			
			**P-3**	**P-2**	**P-1**	**P0**
1	6	2	0	1.99	1.94E2	1.060E3
2	5	2	0	1.5e1	5.98E1	6.86E2
3	1	3	0	0	4.12	3.0E1
4	2	3	0	0	6.57E1	3.15E2
5	9	2	0	3.3	2.58E1	2.78E2
6	5	2	0	9.82	1.58E2	2.150E3
7	11	2	n/a	0	3.48	1.36E2*

*Indicates peak day for mouse 7 based on our obtained data, but no data was available for 4 dpi for this mouse. The regression equation used for predicting day of viremia onset based on the number of infectious mosquitoes feeding is: Day of viremia onset = 2.88 – 0.012x(# infectious mosquitoes).

### Recapitulation of transmission cycle

**Figure 6:**
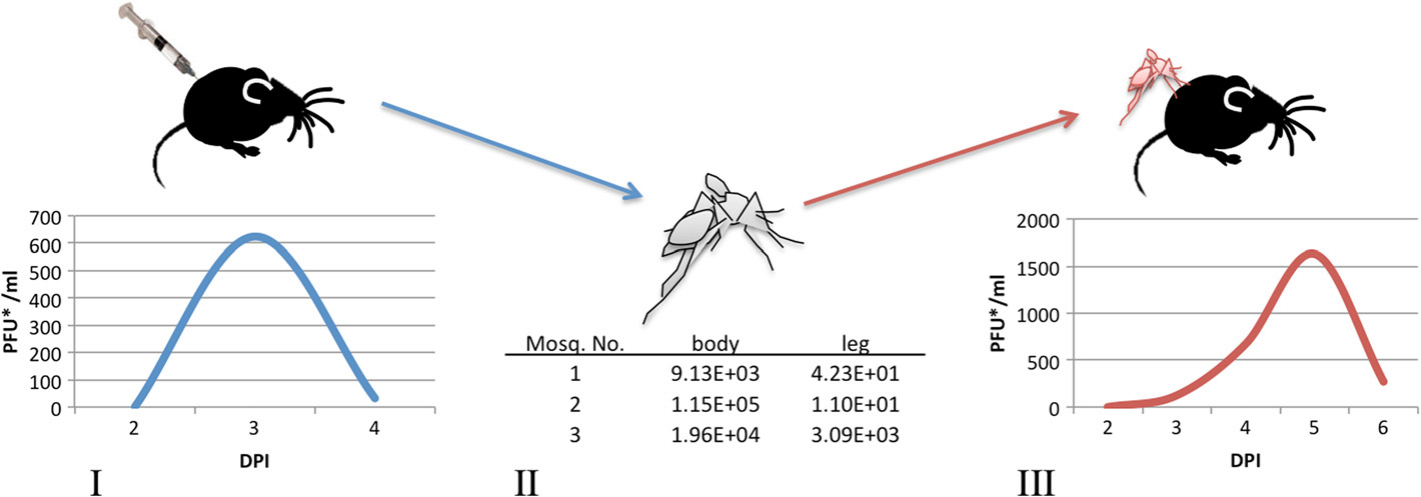
**Recapitulation of the DENV transmission cycles.** On day 3 post infection (dpi), a viremic mouse was fed on by naïve mosquitoes (**I**), resulting in three disseminated infections from naïve mosquito exposure. The resulting body and leg titers from these mosquitoes are shown in the table (**II**). A naïve mouse was then exposed to these mosquitoes 16 dpi and the resulting viremia from that successful transmission is shown (**III**).

Three additional mice were exposed to DENV-2 via needle inoculation in a separate experiment. Naïve mosquitoes then fed on these mice during the resulting viremic period. Engorged mosquitoes were separated and held for 16 days post exposure, before being allowed to re-feed on naïve mice. Immediately afterwards, these mosquitoes were tested for DENV titers in the body and legs. Two out of three mice supported acquisition to mosquitoes. The mean body and leg titers (PFU/mL) of the mosquitoes infected from these mice were as follows for body and leg (range), respectively: 4.78 × 10^4^ (9.1 × 10^3^-1.15 × 10^5^) and 2.23 × 10^3^ (1.1 × 10^1^-7 × 10^3^). One or more of the mosquitoes (n = 3) that acquired DENV from mouse #1 successfully transmitted virus to a naïve mouse, while the attempts with the one infectious mosquito from mouse #2 failed. The viremia curve resulting from that exposure is given in Figure 6.

## Conclusion

We confirmed that the IRF3/7 ^−/− −/−^ mouse is a susceptible vertebrate model for DENV infection and transmission. It has the ability to support non-mouse adapted DENV strains of serotype 2. Though viral loads did not approach those seen in human DENV cases, we confirmed that the virus was actively replicating, and therefore the observed viremia and transmission events were not due to the initial inoculum acting as a contaminant.

Our finding that DENV was no longer detectable following the peak of IFN-γ in these mice reaffirms the role of the IFN-γ response in the clearance of DENV infection [46,47]. This response mirrors the correlation between IFN-γ response and viremia cessation observed in children with DENV [46,48]. The association between IFN-γ response and viremia supports the use of transmission models with intact IFN- γ responses and suggests the potential to use this IRF3/7 ^−/− −/−^ mouse for further inferential efforts to characterize the mechanism of DENV transmission to humans.

Some differences were noted in the IFN-γ response in mosquito exposed versus needle-inoculated mice, but the significance of these differences warrants further study. Studies have shown that interferon-γ-inducible protein 10 (IP-10/CXCL10) is an important chemokine in DENV infection, as DENV resistance has been correlated to increased levels of IP-10 [17,49,50]. More specifically, others have shown that DENV interacts with cell surface heparan sulfate and that IP-10 directly inhibits this interaction and subsequent infection of new cells [51]. This would suggest that delaying the peak of IFN-γ, and thus IP-10, would widen the window for successful replication and infection of new cells. The mechanistic origin of these differences, whether based in mosquito saliva or some intrinsic vertebrate or viral characteristic, requires investigation, but underscores the further importance of a transmission model with an intact IFN-γ response. An important limitation of this particular result is the measurement of systemic cytokine levels. It is likely that a more significant alteration of the vertebrate immune response would be seen at the bite site (due to mosquito salivary secretions) and not necessarily systemically. Therefore, it is important to continue to investigate the potential role of mosquito saliva in infection kinetics in this transmission model.

The finding that viremia was higher in mosquito-inoculated mice compared to needle inoculated mice is unsurprising, given that others have shown similar results with other mosquito borne viruses, such as West Nile [42]. Further, others have shown that the course of dengue infection can be affected if delivered by infectious mosquito bite [35]. It is important to note, however, that mosquitoes in Cox et al. were intrathoracically inoculated with virus, while our mosquitoes were orally challenged, representing a more natural course of virus replication within and subsequent transmission from the mosquito [35]. While caution should be used when comparing the responses of humanized mice to those of non-humanized mice, our results also indicate an enhancement effect of mosquito bite on DENV infection, demonstrating successful DENV transmission with a single infectious mosquito (Table 1).

A significant finding of this study was that this IRF3/7 ^−/− −/−^ mouse strain was able to be both infected by and transmit virus to mosquitoes, thus providing means to replicate the natural transmission cycle of DENV. Completion of the transmission cycle was only successful in 1 out of 2 attempts, where mosquitoes were known to have disseminated infections. Since transmission was achieved in 100% of the other trials (including one other with only 1 infectious mosquito), it may be that the probability of transmission when only one infectious mosquito is feeding is less than 1. Indeed, this is consistent with another study that found consistent results (vRNA levels, clinical signs) only with greater than 4 mosquitoes [35]. The ability of low levels of viremia seen in these infected mice to support transmission to mosquitoes and then subsequent transmission to other naïve mice confirms the presence of actively replicating, viable virus. The acquisition of infection by mosquitoes at these viremia levels suggests important ramifications for the role of low titer human infections (such as might occur in inapparent infections or transitional viremic periods) in overall transmission intensity. Endy, et al. estimated that over 60% of DENV infections in a school cohort were inapparent, and the results from this study point to the need for investigation into the implications for low viremic transmission [52,53].

As there is currently no approved vaccine for DENV, public health monitoring and a greater understanding of transmission dynamics leading to outbreak events are critical for DENV prevention. The ability to achieve successful infection establishment using natural levels of viral inoculum from non-mouse adapted DENV strains is an important step towards studying transmission of DENV [54]. Further, the recapitulation of the transmission cycle in this mouse could provide a tool with which to study the transmission dynamics and kinetics at the mosquito bite site. The further characterization of DENV using this model will expand our knowledge of key entomological, virological and immunological components to infection establishment and transmission events.

## Methods

All experiments met the approval and conditions of the LSU Institutional Animal Care and Use Committee (protocol # 09–077). LSU IACUC procedures and policies adhere to and comply with the guidelines stated in the NIH Guide for the Care and Use of Laboratory Animals. Mice were maintained by laboratory staff and the Department of Laboratory Animal Medicine (DLAM) of the LSU School of Veterinary Medicine.

### Mice

Mice were the generous gift of Dr. M. Diamond (Washington University, St. Louis, MO) with permission from Dr. T. Taniguchi (University of Tokyo, Tokyo, Japan). IRF3/7 ^−/− −/−^ (double knockout) mice are a strain of C57BL/6 mice that lack functional IRF 3 and 7, and thus have a stunted type I IFN response. The type II IFN response and all other immune responses are intact, though diminished [36]. Control mice, wild type C57BL/6 mice, were also included and were obtained from the LSU DLAM.

### Mosquitoes

*Aedes aegypti* (Rockefeller strain) mosquitoes used in this study come from a long-standing colony maintained at the LSU School of Veterinary Medicine. Mosquitoes are reared in constant environmental conditions and density. Mosquitoes have access to sucrose-soaked cotton *ad libitum* until 24 hours prior to experiments.

### Virus

Strain 1232 of DENV serotype 2 (DENV-2 1232) virus was used in these experiments. This strain was originally isolated from a patient in Indonesia in 1978 (personal communication, R. Tesh). Virus was propagated in the C6/36 cell line (*Aedes albopictus* origin) prior to inoculation into mice to avoid adaptation to mammalian cells. Before inoculation, virus was titered via plaque assay on Vero cells and experimental titers from cell supernatant were confirmed at 10^6^ PFU/mL by qRT-PCR as in [55]. Thus, viral titers are expressed as PFU-equivalents/mL, symbolized as PFU*/mL. The resultant virus stock offered to mosquitoes via membrane feeder was passaged in Vero cells, in this case to avoid adaptation to insect cells.

### Virus inoculation and serum collection

Mice (n = 5) were temporarily anesthetized with isoflurane and injected subcutaneously with 100 μl of supernatant from the virus inoculated cell culture. Thus, the total inoculum administered was 10^5^ PFU/mouse of virus. We chose to needle-inoculate mice on the high end of the estimate from Styer 2007 et al. and Cox 2012 et al., so that a potential enhancement by mosquito delivery would not be attributable to a reduced inoculum in the needle group [35,54]. Thus differences are a conservative estimate since mosquitoes may inoculate less than 10^5^ PFU/mL.

Mice were then bled each day for five days via submandibular bleeding technique [56]. Blood was allowed to clot for thirty minutes at room temperature and then centrifuged at 6000 rcf for four minutes. Clarified serum was collected and placed into a clean microcentrifuge tube for analysis.

### Viral detection

vRNA was extracted using the MagMax-96 Total Nucleic Acid isolation kit (Ambion/Life Technologies, Carlsbad, CA) or QIamp Viral RNA Mini Kit (Qiagen, Valencia, CA). Serum samples were brought to volume where necessary with BA-1 diluent and kits were run per manufacturers’ instructions [57]. Detection of vRNA was done using the One-Step TaqMan qRT-PCR assay (Life Technologies, Carlsbad, CA) as noted above [55].

### Verification of viral replication in IRF3/7 ^−/− −/−^ mice

To verify that the mouse model supported active viral replication and that the viremia detected was not simply residual inoculum, we developed a complement strand assay to detect only actively replicating virus. From two mice inoculated with DENV in the same cohort as mentioned above, tissue surrounding the inoculation site and draining lymph nodes were harvested at 48 hours post-inoculation. The primers used amplified the region between nucleotides 1503–2418, covering the envelope protein (1503–2399) and nonstructural protein NS1 (2400–2418): FWD 5′ - GTG CTG CTG CAG ATG GAA GAC AAA - 3′; REV 5′ - TCA CAA CGC AAC CAC TAT CAG CCT - 3′. The reference nucleotide sequence used to generate the primers and from which the protein coding positions are taken is “DENV 2 isolate DENV-2/CO/BID-V3375/2007, complete genome” (GenBank: GQ868558.1). Also included were a positive control of freeze thawed, DENV-2 1232 infected Vero cell culture supernatant, and a negative control of uninfected, age matched Vero supernatant.

Reverse transcriptase (RT)-PCR amplification was carried out using the SuperScript™ III One-Step RT-PCR System with Platinum^®^ Taq DNA Polymerase (Life Technologies, Carlsbad, CA) kit on the DNAEngine Peltier Thermal Cycler (BioRad) with the following protocol. RT Step (1 cycle): 55°C for 30 minutes, 4°C hold (indefinite), 94°C for 2 minutes. At the beginning of the RT step, only the forward primer is included in order to target only the negative sense RNA strand generated during DENV replication. During the 4°C hold, the tubes were removed and a primer mix containing both the forward and reverse primers is added. Tubes were replaced and the run continued. Amplification (40 cycles): 94°C for 15 seconds, 60°C for 30 seconds, 72°C for 1 minute. Final extension and Cool Down (1 cycle): 68°C for 5 minutes, 4°C hold. Samples were then run on a 1% agarose gel and bands visualized (Figure 2).

### Mosquito exposure and transmission to IRF 3/7 ^−/− −/−^ mice

Mosquito inoculated infections occurred in two experimental runs (n_1_ = 7, n_2_ = 3, n_pooled_ = 10). The results from these ten mice were pooled as the “mosquito infected” cohort at LSU School of Veterinary Medicine, where environmental conditions are held steady. Mosquitoes are colony raised, experience consistent rearing conditions, and do not show the phenotypic plasticity seen in field caught mosquito populations.

Mosquitoes were offered an infectious blood meal containing DENV 3–5 days post emergence with an infectious titer of 10^6^ PFU/mL. The blood meal consisted of bovine blood in Alsever’s anticoagulant (Hemostat, Dixon, CA) mixed 2:1 with a virus solution in a total volume of approximately 3 mL per carton of mosquitoes, heated to 37°C and kept warm via the Hemotek device (Discovery Workshops, Arrington, Lancashire, UK). Mosquitoes were allowed to feed for 45 minutes before the blood meal was removed. Each carton consisted of approximately 100 mosquitoes and seven cartons were fed for this study. Engorged females were separated and placed in new cartons and kept at constant environmental conditions (16:8 hours light:dark, 28°C, 70-80% humidity). Approximately 50% of mosquitoes were females, and nearly all fed to repletion before being returned to the aforementioned environmental conditions for nine days of extrinsic incubation. Subsequently, seven mice were anesthetized and placed on top of a carton containing approximately fifty DENV-exposed mosquitoes (1 mouse per carton). After twenty minutes, engorged mosquitoes were separated based on the presence of visible blood in the abdomen. Immediately after feeding on the mice, mosquitoes were frozen, their legs separated from their abdomens, and tested for virus. Testing was done via qRT-PCR on the Roche LightCycler 480 (Roche Diagnostics Corp., Indianapolis, IN) as previously described [55].

### Transmission from viremic mice to mosquitoes

Three mice were inoculated with 10^5^ PFU/mouse of DENV-2 via needle inoculation as previously described. Starting at 48 hours post inoculation, and each day thereafter, mice were anesthetized and placed on top of cartons (1 mouse per carton) containing naïve mosquitoes that had emerged three to four days prior to the beginning of the experiment. Mosquitoes were allowed to feed for approximately twenty minutes and then the mice were removed. Engorged females were separated and placed in clean cartons and kept at constant environmental conditions as described above. Whole mosquito bodies were tested for the presence of virus five days post exposure to determine infection status.

### Cytokine measurement and statistical analysis

IFN-γ, (tumor necrosis factor) TNF-α, (interleukin) IL-4 and IL-1β were measured using the Millipore Milliplex MAP Mouse Cytokine/Chemokine kit as per manufacturer’s instructions (Millipore, Billerica, MA).

Viremia and cytokine levels were statistically analyzed using analysis of variance (ANOVA) on a daily basis. Days of detectable viremia were re-centered around the peak viremia day: three days before peak day (P-3), two days before peak day (P-2), the day before (P-1), peak day (P0), the day after peak (P1) and where relevant, two days after peak (P2). In mosquito inoculated mice, day of viremia onset and peak viremia level relative to the number of mosquitoes was analyzed using simple linear regression. All analyses were performed in SAS 9.13 (PROC MIXED, PROC REG, Carey, NC). Significance is reported at the α = 0.05 level.

### Recapitulation of the transmission cycle

Three mice were inoculated with 10^5^ PFU/mouse of DENV, serum was collected for virus detection daily, and mosquitoes were allowed to feed as described above. Mosquitoes were then held at constant environmental conditions as described above and at sixteen days post exposure, the three cohorts of mosquitoes exposed to the highest viral titers (determined upon testing of mouse serum) were allowed to re-feed upon naïve mice. Immediately after re-feeding, mosquitoes were frozen, legs removed from bodies and tested for infection and dissemination status as in [55]. These mice were subsequently bled daily and serum was collected and tested for DENV presence and titer.

## Competing interests

The authors declare that they have no competing interests.

## Authors’ contributions

RCC and CNM designed the experiments. RCC, MKM, AMJ, and DMC performed the laboratory experiments. RCC performed statistical analyses and wrote the manuscript. MKM, CNM and DMC assisted in editing of the manuscript. All authors read and approved the final manuscript.
